# Relative genomic stability of adipose tissue derived mesenchymal stem cells: analysis of ploidy, H19 long non-coding RNA and p53 activity

**DOI:** 10.1186/scrt529

**Published:** 2014-12-17

**Authors:** Orly Ravid, Ofer Shoshani, Meirav Sela, Ada Weinstock, Tommy Weiss Sadan, Eyal Gur, Dov Zipori, Nir Shani

**Affiliations:** Department of Molecular Cell Biology, Weizmann Institute of Science, 234 Herzl St., Rehovot, 761000 Israel; Department of Plastic Surgery, Tel-Aviv Medical Center, 6 Weizmann Street, Tel-Aviv, 64239 Israel

## Abstract

**Introduction:**

Mesenchymal stem cells (MSCs) are multipotent and have been derived from various tissues. Although MSCs share many basic features, they often display subtle tissue specific differences. We previously demonstrated that bone marrow (BM) MSCs frequently become polyploid in culture. This tendency was mediated by a reduction in the expression of H19 long non-coding RNA during the transition from a diploid to a polyploid state.

**Methods:**

MSCs were derived from both BM and adipose tissue of mice and expanded under normoxic and hypoxic culture conditions. Cells were stained by propidium iodide and their ploidy was evaluated by FACS. Gene expression of independent MSC preparations was compared by quantitative real time PCR and protein expression levels by Western blot analysis. p53 silencing in MSCs was performed by a specific small hairpin RNA (shRNA).

**Results:**

We set to examine whether genomic instability is common to MSCs originating from different tissues. It is demonstrated that adipose derived MSCs (ASCs) tend to remain diploid during culture while a vast majority of BM MSCs become polyploid. The diploid phenotype of ASCs is correlated with reduced H19 expression compared to BM MSCs. Under hypoxic conditions (3% oxygen) both ASCs and BM MSCs demonstrate increased RNA expression of H19 and Vascular endothelial growth factor A. Importantly, ASC gene expression is significantly less variable than BM MSCs under both oxygen conditions, indicating to their superior homogeneity. Gene expression analysis revealed that p53 target genes, often induced by DNA damage, are up-regulated in ASCs under basal conditions. However, p53 activation following treatment with DNA damaging agents was strongly elevated in BM MSCs compared to ASCs. We found that p53 is involved in maintaining the stable diploid state of ASCs as p53 shRNA induced ploidy changes in ASCs but not in BM MSCs.

**Conclusions:**

The increased genomic stability of murine ASCs together with their lower H19 expression and relative homogeneity suggest a tissue specific higher stability of ASCs compared to BM MSCs, possibly due to higher activity of p53. The tissue specific differences between MSCs from a different tissue source may have important consequences on the use of various MSCs both *in vitro* and *in vivo*.

**Electronic supplementary material:**

The online version of this article (doi:10.1186/scrt529) contains supplementary material, which is available to authorized users.

## Introduction

The ability of mesenchymal cells to propagate under tissue culture conditions has been studied since the 1960s. Initially, the capacity of bone marrow (BM)-derived mesenchymal stromal cells to regenerate bone and the BM microenvironment *in vivo* was demonstrated [1]. Mesenchymal cells were then also derived from the stromal vascular fractions of fat tissue and termed preadipocytes [2, 3]. Cultured mesenchymal cells were later termed mesenchymal stem cells (MSCs) to denote their multipotent differentiation potential, and the use of these cells for regenerative medicine was suggested [4]. Since their identification, multipotent MSCs were isolated from most adult tissues, cord blood and placenta [5]. MSCs usually express a series of cell surface markers [6]. However, their definition lies mainly upon their multipotent nature, adhesiveness and morphology [6, 7]. Although MSCs from different organs share many common features, they may also harbor tissue-specific functions. Characterization of the tissue-specific properties of MSCs may assist the understanding of their physiological functions.

Human MSCs have a relatively stable genome at early passages, but may acquire chromosomal aberrations during extended culture [8, 9] and tend to undergo senescence preventing their long-term propagation [9]. Gaining better understanding of the mechanisms that govern genomic stability of MSCs during passaging may allow one to define improved culture conditions that will prevent these events and the tumorigenic transformation of the MSCs. We previously demonstrated that the majority of murine BM MSCs become polyploid in the early stages of their expansion in culture and that the noncoding RNA H19 plays a major part in these events and predicts their corresponding tumor-forming capacity [10].

Here we show that, unlike BM MSCs, MSCs from adipose tissue origin retain their normal diploid state during culture. This tendency is accompanied by a reduced H19 expression compared with similar cells from the BM, further suggesting that a restrained H19 expression preserves the normal diploid state. Importantly, adipose-derived mesenchymal stem cells (ASCs) that were prepared from independent mice also demonstrated a significantly more homogeneous expression phenotype of multiple genes compared with BM MSCs under both normal and stress culture conditions. A partial explanation for the higher stability of ASCs may be provided by their increased basal activation of the p53 signaling pathway, which may help preserve their genomic integrity during passaging.

## Methods

### Cell culture

MSCs were derived from the BM of C57Bl mice (Harlan, Jerusalem, Israel). BM MSCs were flushed from femurs and tibias using murine MesenCult Basal Media and supplement (Stem Cell Technologies, Vancouver, Canada). ASCs were isolated from the mice intra-abdominal fat tissue using 0.1% collagenase (Sigma, Rehovot, Israel) and were separated from fat by centrifugation. Both isolates were expanded under tissue culture conditions as detailed below.

To examine the effect of different cell culture media on the ploidy of ASCs, the cells were cultured either in ADSC Basal Media and supplement (Lonza, Walkersville, MD, USA), murine MesenCult Basal Media and supplement (Stem Cell Technologies) or Dulbecco’s modified Eagle’s medium high glucose with 10% fetal calf serum, 60 μg/ml penicillin and 100 μg/ml streptomycin as stated. For comparison of BM and ASCs from the same mouse donor, all cells were cultured in murine MesenCult Basal Media and supplement; the purity and cell number were high enough to be used experimentally for ASCs at passage 4 while BM MSCs were ready for first derivation at passage 7 or higher.

For hypoxia, cells were cultured in 37°C with 10% carbon dioxide in a Thermo Scientific Forma incubator (Thermo Scientific, Waltham, MA USA) monitored to 3% oxygen from the moment of isolation.

The Weizmann and Tel Aviv Medical Center Institutional Animal Care and Use Committee approved all animal experiments.

### Differentiation

For adipogenesis, confluent cultured cells in a 24-well plate received adipogenic medium containing 10 μg/ml insulin (Sigma), 1 × 10^–6^ M dexamethasone (Sigma) and 0.5 mM IBMX (Sigma). ASC adiopogenic medium contained also 50 μM indomethacin (Sigma). The cells were grown for 3 weeks, with medium replacement twice a week. Adipogenesis was detected by Oil red O staining.

For osteogenesis, confluent cultured BM MSC cells in a 24-well plate received osteogenic medium containing 50 μg/ml l-ascorbic acid-2 phosphate, 10 mM glycerol 2-phosphate disodium salt, and 1 × 10^–8^ M dexamethasone (all from Sigma). For osteogenesis, confluent ASCs were cultured in the StemPro^®^ Osteogenesis Differentiation Kit (Gibco, Grand Island, NY, USA). The cells were grown for 3 weeks with medium replacement twice a week. Osteogenic differentiation was detected by Alizarin red staining. Photographs were taken using an Olympus IX71 microscope equipped with a DP51 camera (Olympus, Tokyo, Japan).

### Flow cytometry

For surface marker analysis, MSCs were harvested and incubated with specific phycoerythrin (PE)-labeled antibodies for 1 hour. Antibodies anti-CD11b–PE, anti-CD45.2–PE, anti-CD31–PE, anti–Ter-119–PE and anti-CD34-PE, rat IgG2b isotype control–PE and rat and mouse IgG2a isotype controls–PE were purchased from eBioscience (eBioscience, San Diego, California, USA), anti–SCA-1–PE was purchased from PharMingen (San Diego, California, USA), and anti–CD34–PE was purchased from Santa Cruz (Santa Cruz, Dallas, Texas, USA). Cells were analyzed using the LSRII flow cytometer (Becton, Dickinson, New Jersey, USA).

For DNA content estimation, cells were fixed with 70% ethanol/phosphate-buffered saline, treated with RNaseA 0.4 mg/ml (Sigma), and stained with propidium iodide 0.1 mg/ml (Sigma). Labeled cells were analyzed using a FACScan flow cytometer (Becton Dickinson Immunocytometry Systems). Fresh splenocytes were used as control diploid cells.

### Chromosome count

Chromosome spreads were prepared from all cells at passage 7 or higher, treated with KCl (0.075 M) and fixed using methanol/acetic acid (3:1), and were then stained with 4′,6-diamidino-2-phenylindole and visualized using a Zeiss Axio Imager Z1 microscope (Carl Zeiss Microscopy, Göttingen, Germany). Chromosomes from at least 10 metaphases were counted from each cell preparation.

### Ultraviolet irradiation

BM MSCs or ASCs (200,000 cells) from similar passages were plated in 6 cm plates. The next day, cells were irradiated at UV-C (254 nm) using a low-pressure mercury lamp (TUV 15w G15T8; Philips, Andover, MA, USA). After 24 hours, cells were taken for RNA analysis.

### Oxidative stress

Oxidative stress was induced by adding hydrogen peroxide (H_2_O_2_; Sigma) for 1 hour at a concentration of 100 μM. RNA extraction was performed 6 hours after the H_2_O_2_ treatment.

### DNA double-strand break induction

DNA double-strand breaks were induced by incubating the cells in 0.5 μg/ml doxorubicin (Sigma) for 24 hours. At the end of the 24-hour incubation, cells were washed and RNA was extracted.

### p53 knockdown

p53 knockdown experiments were carried out in the laboratory of Prof. Varda Rotter, (Weizmann Institute of Science, Rehovot, Israel). The p53 short hairpin RNA vector and its human Rb short hairpin RNA control vector were kindly provided by Dr SW Lowe (Cold Spring Harbor Laboratory, Cold Spring Harbor, NY, USA). Ecotropic Phoenix-packaging cells were transfected with 10 μg DNA of the appropriate retroviral construct by a standard calcium phosphate procedure. Culture supernatants were collected 36 to 48 hours after transfection and filtered. BM MSCs and ASCs were infected with the filtered viral supernatants in the presence of 4 μg/ml polybrene (Sigma) for 12 hours, after which the medium was changed. Fresh viral suspensions were added after a 24-hour interval for an additional 12 hours. RNA was extracted from the cells to verify p53 knockdown. The cells were expanded for an additional three passages and then taken for ploidy analysis.

### Western blot

For immunoblotting, proteins were separated by SDS–PAGE, transferred to a nitrocellulose membrane and detected with the anti-p53 antibody (Santa Cruz) or primary antibody or an anti-glyceraldehyde-3-phosphate dehydrogenase (Sigma) and horseradish peroxidase-conjugated secondary antibody using enhanced chemiluminescence western blotting reagents (Thermo Scientific) and film (Fujifilm, Tokyo, Japan). Densitometry was conducted using ImageJ software ().

### Quantitative real-time PCR

RNA from passage 4 ASCs and passage 7 or higher BM MSCs was extracted using the NucleoSpin RNA II kit (Macherey-Nagel, Düren, Germany), and cDNA was prepared using M-MLV Reverse Transcriptase (Promega, Madison, WI, USA) according to the manufacturer’s protocols. Real-time PCR was carried out using the perfeCTa SYBR mix (Quanta BioSciences, Gaithersburg, MD, USA) and processed using Step One Plus (Applied Biosystems Foster, CA, USA) with normalization to HPRT or 18S.

The real-time primers used are as follows: Hif1α, 5′-ACAAGTCACCACAGGACAG-3′ and 5′-AGGGAGAAAATCAAGTCG-3′ [11]; VEGFa, 5′-GCGGATCAAACCTCACCAAA-3′ and 5′-TTCACATCTGCTGTGCTGTAGGA-3′ [12]; H19, 5′-GCTAGGGTTGGAGAGGAATGG-3′ and 5′-AAAAGTAACCGGGATGAATGTCTG-3′ [10]; p53, 5′-AGAGTATTTCACCCTCAAGATCCG-3′ and 5′-CGGAACATCTCGAAGCGTTT-3′; p21, 5′-CCATGAGCGCATCGCAATC-3′ and 5′-CCTGGTGATGTCCGACCTG-3′; btg2, 5′-ATGAGCCACGGGAAGAGAAC-3′ and 5′-GCCCTACTGAAAACCTTGAGTC-3′; Ercc5, 5′-TGCTGGCCGTGGATATTAGC-3′ and 5′-GCCGGTGGAATAATGTGAGAAGA-3′; Mgmt, 5′-TGCTCTCCATCACCCTGTGTT-3′ and 5′-AACACCTGTCTGGTGAATGAATCTT-3′; Puma, 5′-GCGGCGGAGACAAGAAGA-3′ and 5′-TGTGATGATGGTGAGGATGG-3′; Cycling1, 5′-ACAACTGACTCTCAGAAACTGC-3′ and 5′-CATTATCATGGGCCGACTCAAT-3′; Bax, 5′-TGAAGACAGGGGCCTTTTTG-3′ and 5′-AATTCGCCGGAGACACTCG-3′; Mdm2, 5′-TGTCTGTGTCTACCGAGGGTG-3′ and 5′-TCCAACGGACTTTAACAACTTCA-3′; p19, 5′-GGTCGCAGGTTCTTGGTCAC-3′ and 5′-CGGGATCGCACGAACTTCAC-3′; p16, 5′-TTGGGCGGGCACTGAATCTC-3′ and 5′-AGTCTGTCTGCAGCGGACTC-3′; HPRT, 5′-GCAGTACAGCCCCAAAATGG-3′ and 5′-GGTCCTTTTCACCAGCAAGCT-3′; and Rn18s, 5′-CGAAAGCATTTGCCAAGAAT-3′ and 5′-AGTCGGCATCGTTTATGGTC-3′.

### Statistical analysis

Statistical analysis was done using R statistical software and SigmaPlot, v.11 (SigmaPlot, v.11 (Systat Software, San Jose, CA)). *P* <0.05 was considered statistically significant using the Mann–Whitney test or *t* test as stated. The *F*-variance test was used to compare the variance of two groups, *P* <0.05 showing statistical difference.

## Results and discussion

### Unlike BM MSCs, ASCs retain their diploid state under various culture conditions

**Figure 1:**
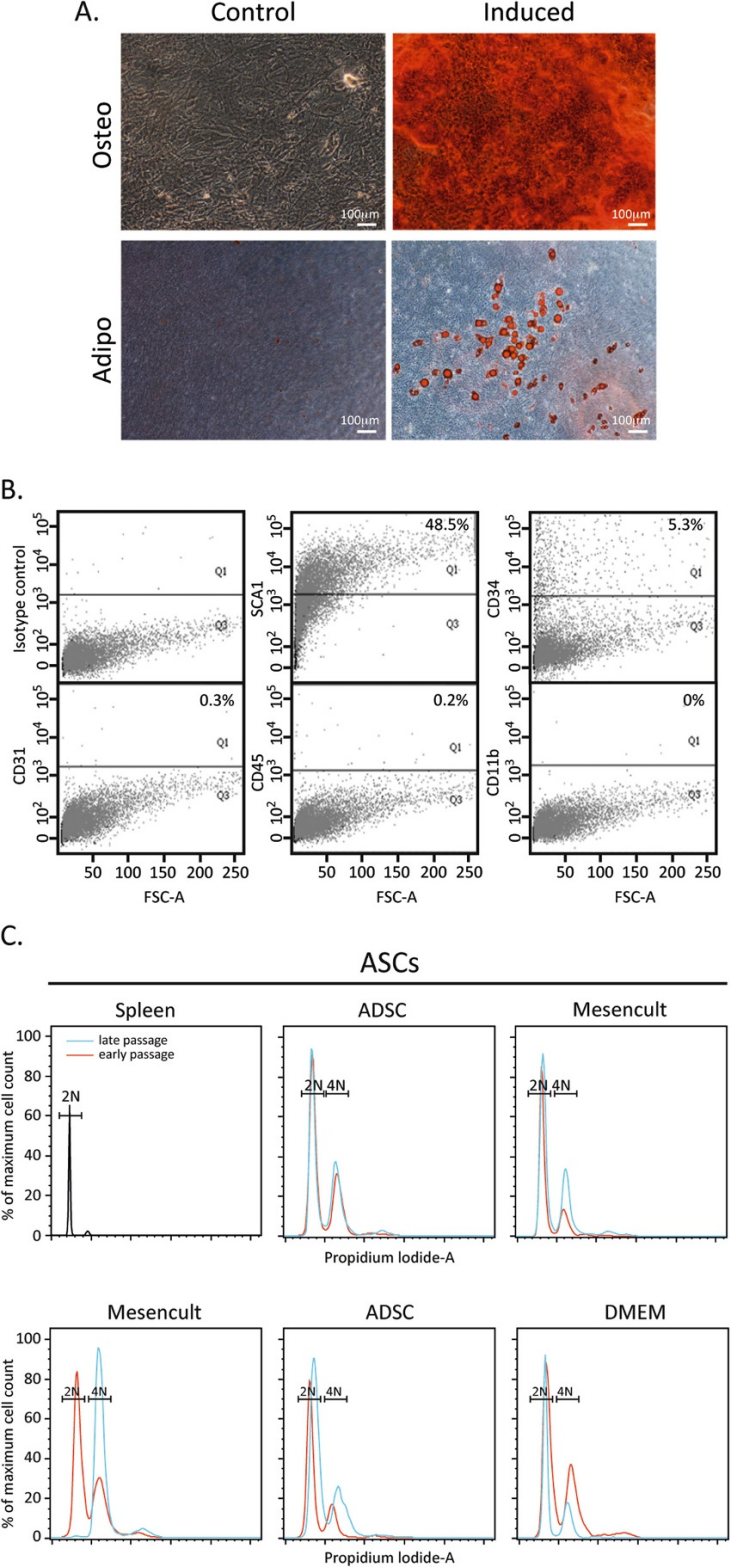
**Adipose-derived mesenchymal stem cells remain diploid under various culture conditions.** Adipose-derived mesenchymal stem cells (ASCs) were characterized according to **(A)** their differentiation potential and **(B)** their surface marker expression (percentage reflects cell-staining above background). **(C)** Five adipose-derived mesenchymal stem cell preparations were grown in different growth media (as indicated: two preparations in Mesencult medium, two preparations in ADSC medium and one preparation in Dulbecco’s modified Eagle’s medium (DMEM)) and their DNA content was analyzed by flow cytometry at early and late passages, diploid (2N) and tetraploid (4N). Primary diploid spleen cells used as control.

**Figure 2:**
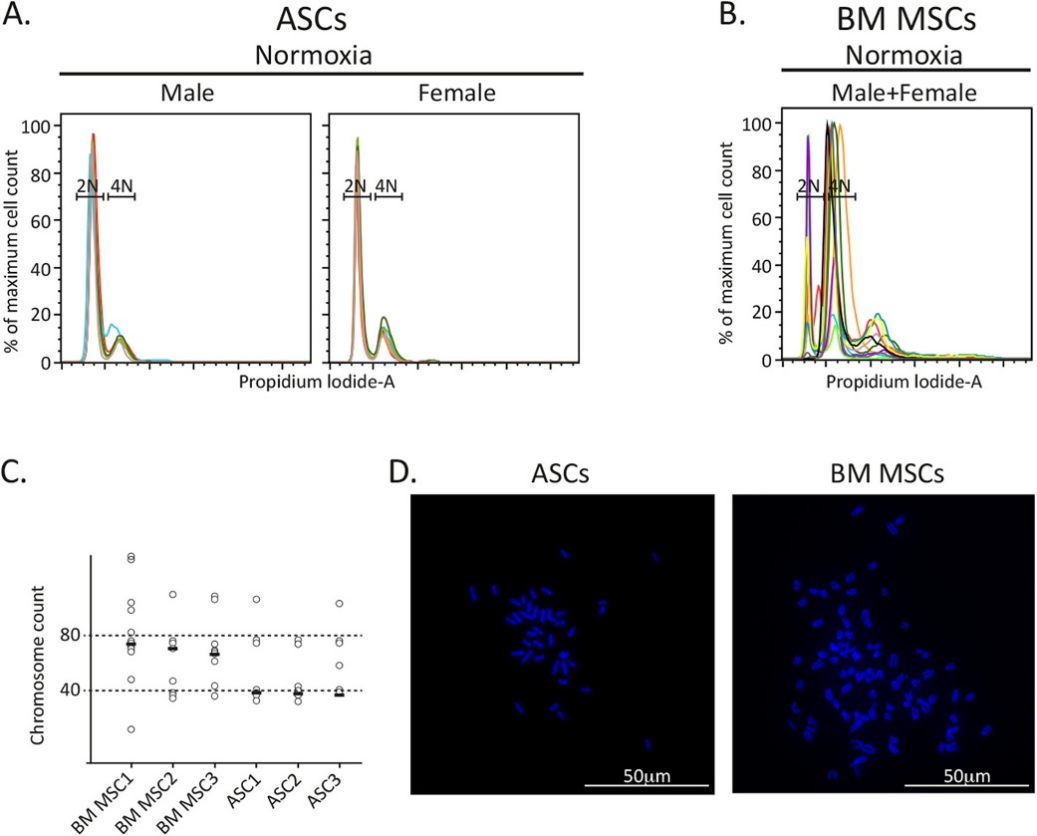
**Cultured adipose-derived mesenchymal stem cells retain their diploid state, in contrast to bone marrow mesenchymal stem cells that become polyploid.** Independent preparations of paired **(A)** adipose-derived mesenchymal stem cells (ASCs) and **(B)** bone marrow mesenchymal stem cells (BM MSCs; prepared from the same male or female mouse), were expanded in Mesencult and their DNA content was analyzed by flow cytometry, diploid (2N) and tetraploid (4N). Resulting plots from male/female ASCs/BM MSCs were overlaid and are presented in a single graph; six female and six male adipose-derived mesenchymal stem cells and six female and five male BM MSCs were used. **(C)** Chromosome spreads were made from three adipose-derived mesenchymal stem cells and three BM MSC preparations and a graphic summary of the median (straight short line) chromosome count is shown (10 to 15 different metaphases were counted from each cell preparation). Horizontal dashed lines, diploid or tetraploid DNA contents at chromosome numbers 40 and 80, respectively. **(D)** Examples of the 4',6-diamidino-2-phenylindole-stained chromosomes from a single preparation of adipose-derived mesenchymal stem cells and BM MSCs as summarized in the previous section.

We have reported previously that mouse BM MSCs frequently become polyploid during culture [10] and wanted to determine whether this tendency is common also to ASCs. All ASC preparations (ASC adipogenic and osteogenic differentiation and surface marker expression characteristics are demonstrated in Figure 1A,B, respectively), regardless of the culture conditions used, were diploid at early passages (passages 3 to 4) and only one out of the five preparations became tetraploid at a later passage (passages 7 to 9) (Figure 1C). Isolates of paired BM MSCs and ASCs (both derived from the same mouse) were extracted from several independent mice (BM MSC adipogenic and osteogenic differentiation and surface marker expression characteristics are presented in Additional file 1). Figure 2A,B shows an overlay of the DNA content of MSC preparations from paired adipose or BM tissue. While the vast majority of ASCs were diploid, most BM MSCs were polyploid with varying amounts of DNA content. The DNA content determination by flow cytometry was confirmed by a chromosome spread analysis, and the median of chromosome number of ASCs and BM MSCs verified their diploid and tetraploid state respectively (Figure 2C,D). ASCs thus demonstrate relative genomic stability under various conditions and in a sex-independent manner.

**Figure 3:**
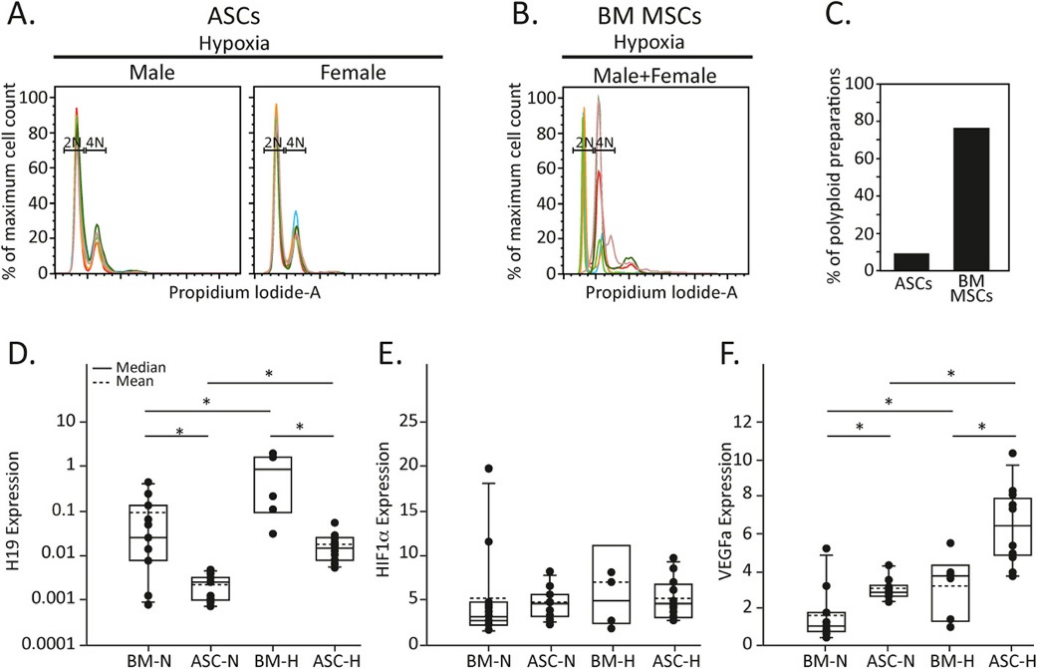
**Independent adipose-derived mesenchymal stem cell preparations retain their diploid state under hypoxia and react homogeneously to changing oxygen conditions.** Independent preparations of paired **(A)** adipose-derived mesenchymal stem cells (ASCs) and **(B)** bone marrow mesenchymal stem cells (BM MSCs; prepared from the same male or female mouse) were expanded in Mesencult under hypoxic conditions (3% oxygen) and their DNA content was analyzed by flow cytometry. Resulting plots from male/female adipose-derived mesenchymal stem cells/BM MSCs were overlaid and are presented in a single graph, diploid (2N) and tetraploid (4N); six female and six male adipose-derived mesenchymal stem cells and six total female and male BM MSCs were used. **(C)** Graph summarizing the percentage of adipose-derived mesenchymal stem cells and BM MSCs that became polyploid out of all cell preparations cultured during the study. **(D)**, **(E) (F)** Comparison of the RNA level of independently derived adipose-derived mesenchymal stem cells or BM MSCs that were expanded either in hypoxic (H) or normoxic (N; normal oxygen) conditions made by quantitative real-time PCR. *P <0.05, Mann–Whitney test (each group size is mentioned in Table 1). HIF1α, hypoxia-inducible factor 1α; VEGFa, vascular endothelial growth factor A.

It was previously reported that MSCs expanded under hypoxic conditions (1 to 5% oxygen) are superior in their culture expansion, differentiation, and genomic stability compared with MSCs that were grown under normoxic conditions (atmospheric oxygen level) and are therefore more suitable for clinical use [13–15]. Figure 3A,B shows an overlay of the DNA content of MSC preparations from paired adipose or BM tissue grown under hypoxic conditions. ASCs grown under hypoxic conditions showed a diploid phenotype while BM MSC preparations demonstrated a polyploid phenotype. Thus, culturing BM MSCs under hypoxic conditions did not significantly inhibit the transition of BM MSCs to a polyploidy state. Altogether, 76% (13 out of 17 cell preparations) of mouse BM MSC preparations, regardless of culture conditions, demonstrated a polyploid phenotype compared with only 9% polyploidy (three out of 32 cell preparations) of ASCs (Figure 3C).

### ASCs ability to retain a diploid state is accompanied by reduced H19 expression

Our previous findings demonstrated that rare diploid BM MSC populations express far higher levels of H19 RNA as compared with polyploid BM MSC and are more tumorigenic [10]. Since most BM MSCs became polyploid, we assumed that the high H19 expression in diploid BM MSCs promotes their instability. It was thus hypothesized that genomic stability would correlate with lower levels of H19. Evaluation of the H19 expression demonstrated that its mean expression in ASCs under normoxic conditions was 43-fold lower than in polyploid BM MSCs, indicating a more stable state of these cells in culture (Figure 3D and Table 1). As shown previously, the H19-IGF2 locus is epigenetically controlled and is important for stem cell function [16]. Our results indicate that there might be a difference in the epigenetic control of H19 expression between ASCs and BM MSCs, as suggested by our RNA expression analysis. Such a difference might reveal additional inconsistencies in the properties of the two cell types, possibly through mir675 expression (which is derived from H19). As the receptor for IGF2, IGF1r, is regulated by mir675, it would be of interest to examine IGF2 signaling in these cells. Future studies characterizing the differentially methylated region of the H19-IGF2 locus in ASCs and BM MSCs are thus required in order to obtain a more broad description of their different properties.

**Table 1:** **Statistical analysis of RNA expression levels comparing groups of different mesenchymal stem cell preparations**

	Mean adipose	Mean BM	Adipose ( ***n*** )	BM ( ***n*** )	***P*** , Mann–Whitney	***P*** , ***F*** -variance
Normoxic adipose vs. BM						
H19	0.002	0.092	11	11	0.004	0
HIF1α	4.786	5.275	11	11	>0.05	0.0007
VEGFa	2.974	1.647	10	11	0.01	0.01
Hypoxic adipose vs. BM						
H19	0.018	0.85	12	6	0.001	0
HIF1 α	5.157	7.078	12	6	>0.05	0.002
VEGFa	6.413	3.227	12	6	0.013	>0.05
	**Mean normoxia**	**Mean hypoxia**	**Normoxia (** ***n*** **)**	**Hypoxia (** ***n*** **)**	***P*** **, Mann–Whitney**	
Normoxic vs. hypoxic						
H19 BM	0.092	0.85	11	6	0.024	
VEGFa BM	1.647	3.227	11	6	0.05	
H19 adipose	0.002	0.018	11	12	0.00005	
VEGFa adipose	2.974	6.413	10	12	0.00015	
Diploid vs. polyploid						
H19 diploid adipose	0.002	0.018	11	12		
H19 polyploid BM	0.099	1.068	10	3		
Polyploid BM/diploid adipose	43.137	59.447				

BM, bone marrow; HIF1α, hypoxia-inducible factor 1α; VEGFa, vascular endothelial growth factor A.

### ASCs response to different oxygen conditions is more homogeneous than that of BM MSCs

H19 expression and vascular endothelial growth factor A expression were shown previously to increase under hypoxic conditions in a hypoxia-inducible factor 1α-dependent manner [17–19]. This was therefore used to compare the responses of BM MSCs and ASCs to different oxygen levels. We found a significant increase in H19 and vascular endothelial growth factor A expression, seen in both ASCs and BM MSCs grown under hypoxic conditions (Figure 3D,F respectively and Table 1). A previous report stated that H19 is elevated in hypoxia only in p53 mutant or null settings [19]. Hypoxia-inducible factor 1α mRNA expression remained unchanged under both oxygen conditions (Figure 3E), most probably since its protein levels are increased mainly due to hypoxia-induced stabilization, rather than to increased transcription [17, 18]. Interestingly, H19 mean expression in ASCs under hypoxic conditions was 59-fold lower than in polyploid BM MSCs, indicating again a more stable state of these cells in culture (Table 1). Indeed, ASCs maintain their diploid state during passaging (Figure 1C).

Although the change in RNA expression under different oxygen levels showed a similar trend in both ASCs and BM MSCs (Figure 3D,E,F), the variation in expression was significantly lower in ASCs compared with BM MSCs, as evident from the *P* (variance) analysis (Table 1), indicating a more homogeneous nature of ASC-independent preparations.

### ASCs ability to retain a diploid state is accompanied by a higher basal activity of p53, as indicated by the higher expression of its downstream targets

**Figure 4:**
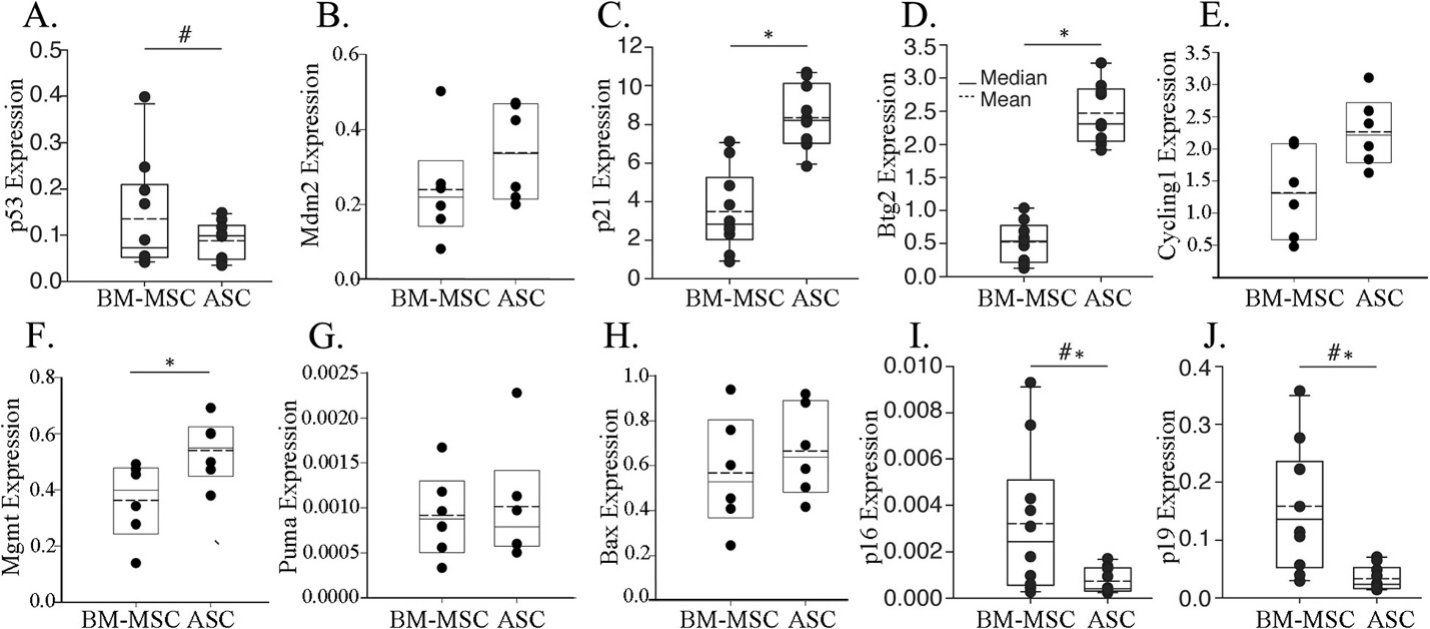
**Adipose-derived mesenchymal stem cells have higher levels of DNA damage-related p53 targets, similar levels of Bcl-2 family p53 targets, and a lower p16 and p19 expression than bone marrow mesenchymal stem cells.** Independent nine to 10 preparations **(A, C, D, I, J)** or six preparations **(B, E, F,** **G, H)** of paired adipose-derived mesenchymal stem cells (ASCs) and bone marrow mesenchymal stem cells (BM-MSCs; prepared from the same male or female mouse) were analyzed for RNA levels of the indicated gene by quantitative real-time PCR. *P <0.05, paired two-tailed *t* test of adipose-derived mesenchymal stem cells vs. BM-MSCs; #*P* <0.05, *F*-variance test of adipose-derived mesenchymal stem cells vs. BM-MSCs.

Normal cells expressing wildtype p53 were demonstrated previously to arrest in the G1 phase subsequent to the induction of tetraploidy. p53 is thus considered to be a guardian of genomic stability that prevents the development of polyploidy in normal cells [20, 21]. Our findings repeatedly show that tetraploid BM MSCs continue to expand and do not undergo a growth arrest although they express wildtype p53 (the wildtype RNA sequence of p53 in tetraploid BM MSCs was examined and verified) (data not shown). A comparison of the RNA expression levels of p53 between BM MSCs and ASCs showed no significant difference (Figure 4A). Consistent with our previous RNA assessments, however, the p53 expression profile was significantly less variable between ASC preparations compared with BM MSC preparations. Similarly to the RNA expression analysis, only a slight increase in p53 protein expression that was not statistically significant was observed in ASCs compared with BM MSCs (data not shown). Being a transcription factor, p53 activity is often determined by its ability to induce the transcription of various target genes. Interestingly, the expression levels of five downstream targets of p53 known to be induced by DNA damage, Mdm2, p21, btg2, Cycling1, and Mgmt was higher in ASCs compared with BM MSCs (Figure 4B,C,D,E,F), indicating a higher basal p53 activity in ASCs. p53 activation in ASCs, however, does not seem to promote the Bcl-2 apoptotic pathway because no increased expression of Puma and Bax pro-apoptotic genes was observed compared with BM MSCs (Figure 4G,H). The lower basal activity of p53 in BM MSCs provides a possible mechanistic explanation for the continuous propagation of BM MSCs following their polyploidization. The higher p53 activity of ASCs may prevent polyploidization, thereby allowing them to retain their genomic stability and a diploid state. As suggested for the H19-IGF2 locus, the differences in p53 activation could be attributed to epigenetic control in general, and gene imprinting specifically. In fact, we have previously established a critical role for epigenetic modulation in the plasticity of BM MSCs [22]. An example is p57kip2, a paternally imprinted gene, which was demonstrated to play a role in cell cycle progression and DNA damage response is well established [23]. Thus, it would be of interest to examine whether this protein and its regulation also play a role in the differences observed between ASCs and BM MSCs.

### Lower expression of p16 and p19 in ASCs compared with BM MSCs

The p19^ARF^ and p16^ink4a^ proteins are important tumor suppressors [24]. In contrast to p21 and btg2, both p16 and p19 expression levels were higher in BM MSCs compared with ASCs (Figure 4I,J). The higher p53 activity of ASCs suggests a higher stability of p53 in these cells. Activation of p53 by the p19 pathway occurs through the interaction of p19 with mdm2, thereby preventing p53 degradation [25]. The higher expression of p19 combined with the lower p53 activation in BM MSCs suggests that p53 activation does not occur in these cells in a p19-dependent manner. This is in accordance with reports suggesting p19 activation and growth arrest in response to oncogene-induced proliferation and not tetraploidization or DNA damage [24, 26, 27]. In agreement with our previous findings, p19 and p16 showed a more heterogeneous expression in BM MSC preparations compared with ASCs.

### Both BM MSCs and ASCs respond normally to DNA damage, as indicated by an increased expression of p53 targets

**Figure 5:**
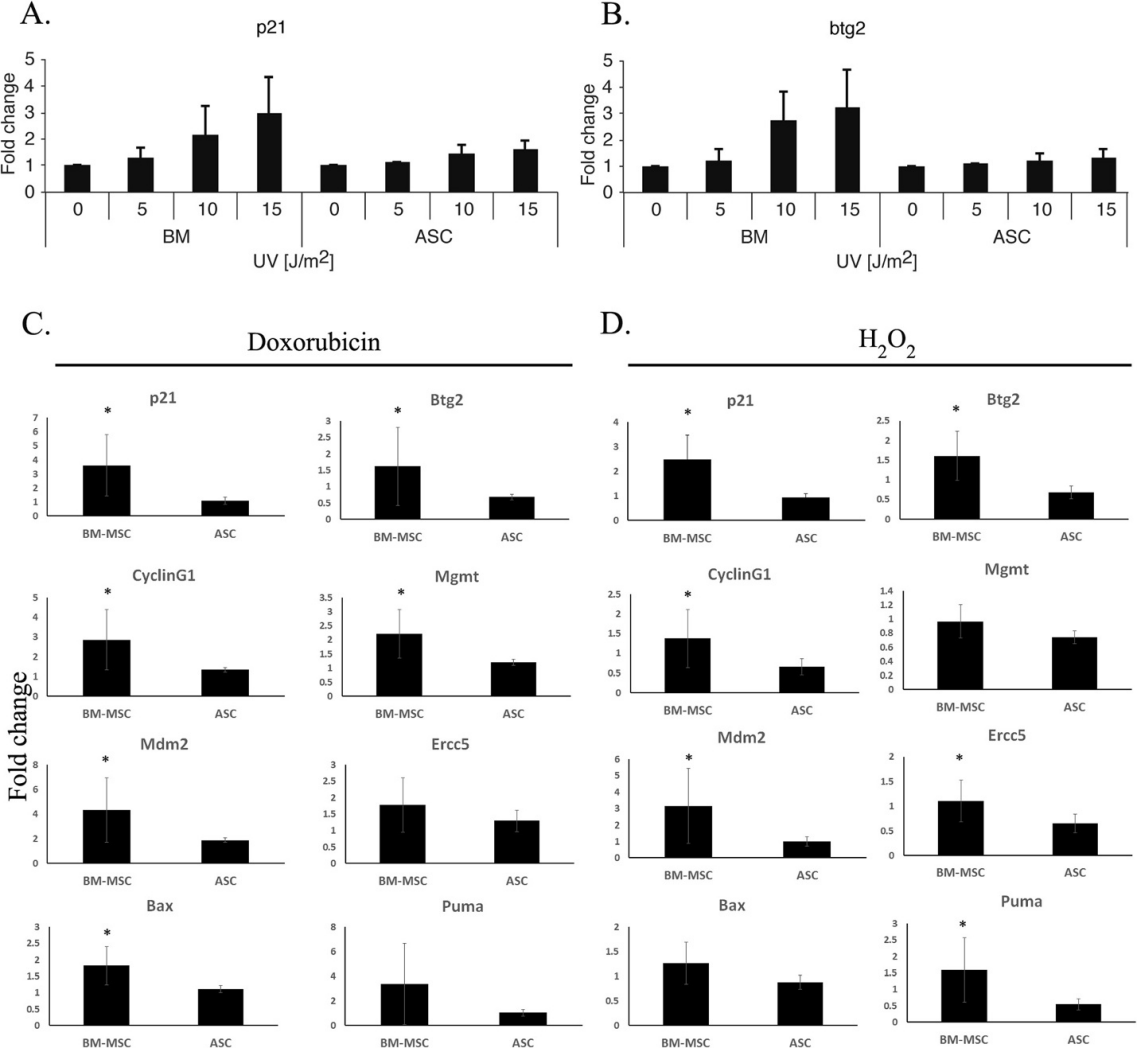
**Adipose-derived mesenchymal stem cells demonstrate milder response to ultraviolet and doxorubicin-induced DNA damage and to oxidative stress. (A)**, **(B)** p21 and btg2 RNA expression after ultraviolet (UV) irradiation in elevating doses (J/m^2^) in four bone marrow mesenchymal stem cell (BM MSC) preparations and three adipose-derived mesenchymal stem cell (ASC) preparations. No irradiation was normalized as 1, bars indicate mean ± standard deviation. Regression of gene expression fold-change as a function of UV intensity was compared between tissues using analysis of covariance, with tissue as a categorical factor, and UV intensity as a covariate. **(C)**, **(D)** Induction of RNA expression of the indicated genes following incubation with either 0.5 μg/ml doxorubicin for 24 hours or 6 hours following incubation with hydrogen peroxide (H_2_O_2_), respectively, was compared between six adipose-derived mesenchymal stem cell and BM MSC preparations. *P <0.05, unpaired equal variance two-tailed *t* test of adipose-derived mesenchymal stem cells vs. BM-MSCs.

Given the difference in p53 activity between ASCs and BM MSCs under basal culture conditions, we set to examine the function of the p53 pathway following ultraviolet-induced DNA damage. Both ASCs and BM MSCs responded in a dose-dependent manner by elevating p53 target genes, demonstrating normal functionality of p53 (Figure 5A,B). The normal p53 DNA damage response further suggests that the continued propagation of BM MSCs in a tetraploid state occurs in the presence of wildtype p53. The extent of the response was greater in BM MSCs compared with ASCs (significantly for btg2 by regression analysis). To confirm the increased responsiveness of BM MSCs to DNA damage signals, we compared the expression levels of various p53 target genes following doxorubicin and H_2_O_2_ exposure. Similarly to ultraviolet, exposure to both doxorubicin and H_2_O_2_ resulted in greater induction of eight p53 target genes (p21, Btg2, cycling1, Mgmt, Mdm2, Ercc5, Bax and Puma) in BM MSCs compared with ASCs, confirming the increased responsiveness of BM MSCs to DNA damage signals (Figure 5C,D). This indicates that ASCs are less responsive to DNA damage, possibly because of their higher p53 activation level.

### ASCs undergo ploidy shifts following p53 depletion

**Figure 6:**
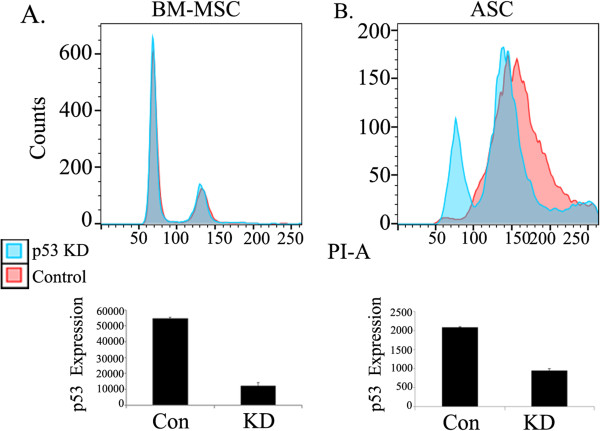
**p53 silencing promotes a ploidy change in adipose-derived mesenchymal stem cells but not in bone marrow mesenchymal stem cells.** Ploidy and p53 expression were evaluated in **(A)** bone marrow mesenchymal stem cells (BM MSCs) and **(B)** adipose-derived mesenchymal stem cells (ASCs) following p53 silencing by a specific p53 short hairpin RNA (shRNA) or a nonrelevant shRNA control. RNA was extracted 3 days after infection. For ploidy analysis, cells were expanded for an additional three passages. Con, control; KD, knockdown.

To examine the role of p53 in maintaining the stable diploid state of ASCs, we depleted p53 mRNA in ASCs and BM MSCs by short hairpin RNA. As can be seen in Figure 6, p53 depletion induced a clear ploidy change in ASCs but not in BM MSCs. This, together with the significant differences found in the activity of p53 between ASCs and BM MSCs under basal and perturbed conditions, suggests that p53 is pivotal in maintaining the diploid state of ASCs.

## Conclusions

The findings presented in this study clearly demonstrate the higher stability of murine ASCs compared with BM MSCs, as reflected in their ability to maintain a diploid phenotype, together with their uniform nature. Importantly, these markedly different phenotypes do not result from different culture conditions (BM MSCs and ASCs extracted from the same mouse were grown under the exact same conditions) but from a signature that most probably comes from their tissue origin. Consistent with our previous report that demonstrated H19 as a major contributor to BM MSC instability and tumorigenic potential, we find here that the increased stability of ASCs is correlated with their significantly lower H19 expression compared with BM MSCs. This further suggests that a restrained H19 expression serves as a stabilizing parameter in cultured MSCs. In addition to their genomic stability, ASCs from independent preparations demonstrated a far more homogeneous expression pattern of multiple genes under normal and stress conditions compared with BM MSCs, indicating that their transcription state is more stable. Interestingly, the superior uniformity and stability of murine ASCs was also correlated with an increased basal p53 activity. The significant difference in p53 activity between ASCs and BM MSCs was also evident following DNA damage (ultraviolet irradiation, H_2_O_2_ and doxorubicin) in which a significantly more robust induction of p53 target genes was seen in BM MSCs. Knockdown of p53 in ASCs resulted in significant ploidy changes, confirming its critical role in maintaining a stable diploid state.

Altogether our results suggest that MSCs isolated from different tissue sources hold distinct properties, which are manifested *in vitro.* This tissue-specific phenotype may have important bearing on the ability of MSCs to reach any intended clinical purpose. Further emphasis should thus be given to elucidating the origin-specific nature of MSCs.

## Electronic supplementary material

Additional file 1: **is Figure S1 showing that BM MSCs express Sca-1 and differentiate into osteocytes and adipocytes.** Upper panel: MSCs were cultured with or without induction media for 2 to 3 weeks to induce cell differentiation. Differentiation into bone was detected by Alizarin red staining. Differentiation into fat was detected by Oil red O staining. Lower panel: MSCs were stained with antibodies against surface markers or control antibodies and subjected to flow cytometry analysis. Each graph represents staining with antibody against a surface marker, nonspecific antibody of the same isotype as control. Percentage reflects % cells stained above background. (TIFF 5 MB)

## Abbreviations

ASC: adipose-derived mesenchymal stem cell
BM: bone marrow
H_2_O_2_: hydrogen peroxide
MSC: mesenchymal stem cell
PE: phycoerythrin.
