# Supplementary figures and images for: Relative genomic stability of adipose tissue derived mesenchymal stem cells: analysis of ploidy, H19 long non-coding RNA and p53 activity
Source: Stem Cell Res Ther. 2014 Dec 17;5(6):139. doi: 10.1186/scrt529 (PMC4446078; doi:10.1186/scrt529)

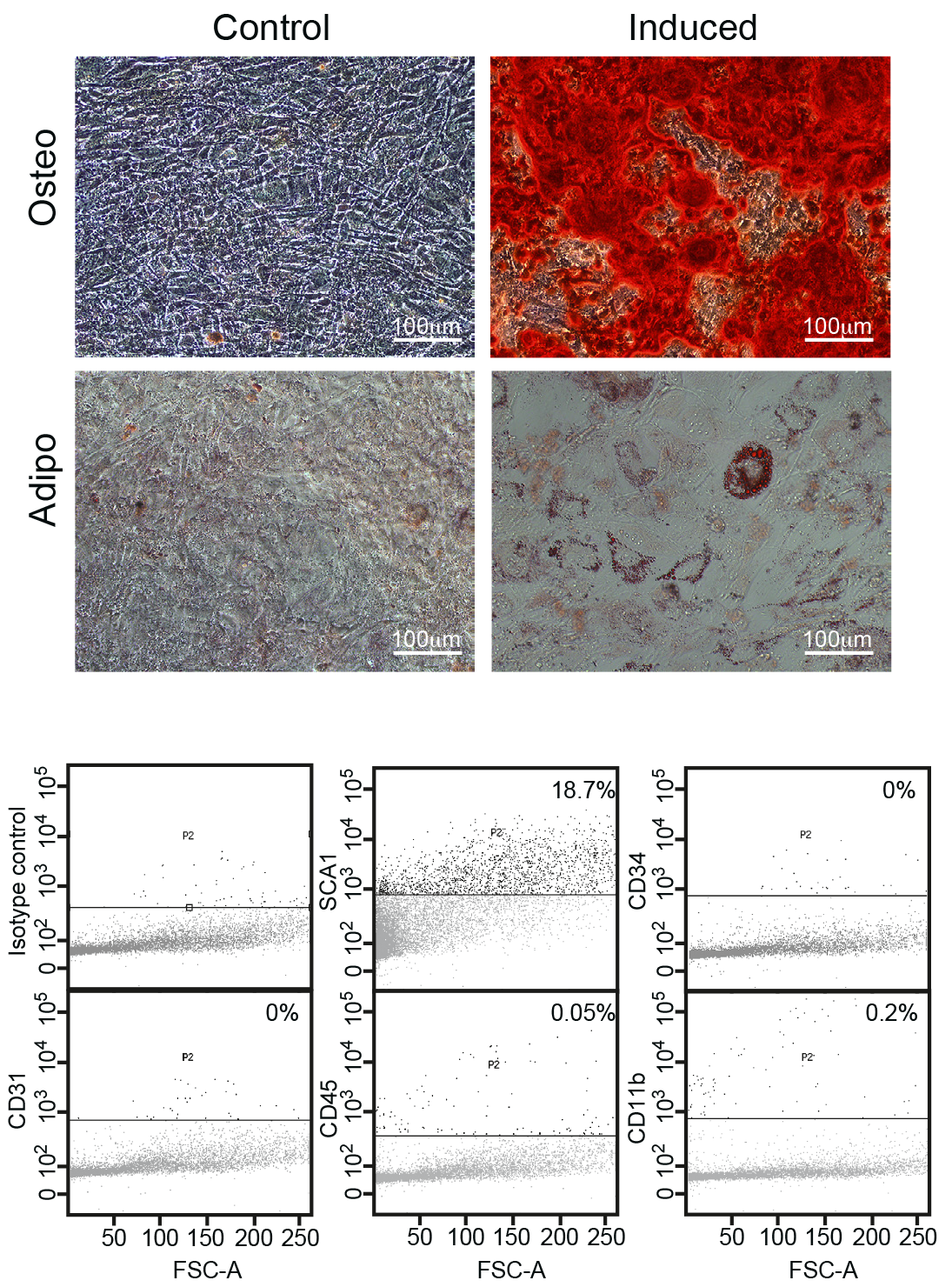

Supplement: Supplementary file 1 — Additional file 1: is Figure S1 showing that BM MSCs express Sca-1 and differentiate into osteocytes and adipocytes. Upper panel: MSCs were cultured with or without induction media for 2 to 3 weeks to induce cell differentiation. Differentiation into bone was detected by Alizarin red staining. Differentiation into fat was detected by Oil red O staining. Lower panel: MSCs were stained with antibodies against surface markers or control antibodies and subjected to flow cytometry analysis. Each graph represents staining with antibody against a surface marker, nonspecific antibody of the same isotype as control. Percentage reflects % cells stained above background. (TIFF 5 MB) [file 13287_2014_447_MOESM1_ESM.tiff]
